# Transcription Factor HSF1 Suppresses the Expression of Surfactant Protein D in Cells Infected with *Aspergillus fumigatus*

**DOI:** 10.3390/pathogens10060709

**Published:** 2021-06-06

**Authors:** Sung-Su Kim, Kwang-Soo Shin

**Affiliations:** 1 Department of Biomedical Laboratory Science, Daejeon University, Daejeon 34520, Korea; 2 Department of Microbiology, Graduate School, Daejeon University, Daejeon 34520, Korea

**Keywords:** aspergillosis, *Aspergillus fumigatus*, HSF1, surfactant protein D

## Abstract

Aspergillosis is a life-threatening disease in patients with compromised immune systems. The process of fungal invasion is an important step during host cell infection. We investigated the transcription factor and promoter region of SFTPD, which is activated during the infection process in conidia-treated cells. To investigate the promoter activity of SFTPD in fungal-infected cells, we cloned various lengths of the promoter region (−1000 to +1) of SFTPD and examined its activity in A549 cells treated with *Aspergillus fumigatus* conidia. We determined the location within the promoter region of SFTPD that exhibits a response to conidia infection. AliBaba 2.1 software was used to predict the transcription factor involved as well as the binding sites in the SFTPD promoter region. The results of a decoy assay show that the HSF1 transcription factor is sufficient to decrease the SFTPD expression. Using chromatin immunoprecipitation, we confirmed that HSF1 directly binds to the selected sequence, which is located in the response region (−142 to −134 bp). These findings suggest that inhibiting the binding of HSF1 to the promoter region of SFTPD is an important step to prevent conidia infection.

## 1. Introduction

Invasive fungal infections are uncommon in immunocompetent individuals, whereas immunocompromised patients run the risk of morbidity and mortality from these diseases. Medical advances have increased the survival of chronically immunosuppressed patients, but the downside of this progress is an increasing number of patients at risk for fungal infections [1,2]. Invasive aspergillosis is a life-threatening condition to those with pulmonary or systemic infections. According to the health of the infected human, *Aspergillus* can result in diseases ranging from allergic reactions in the topical area to invasive infections or even pneumonia. In recent publications, several other risk factors were identified, such as systemic steroid treatment, intensive care unit admission, and influenza [3,4,5,6]. The invasion process is a key step in the infection of human hosts by *Aspergillus fumigatus* [7,8]. *A. fumigatus* is classified as an opportunistic pathogen that is ubiquitous in the natural environment [7].

Pulmonary surfactant proteins exist on lung alveolar surfaces, consist of phospholipids and proteins, and are produced by alveolar type II cells and Clara cells, which are the major types of peripheral airway cells. Surfactant proteins play a key role as a defense mechanism in humans. They reduce tension to prevent an alveolar collapse, inhibit pathogens from spreading, and regulate immune reactions [9]. Pulmonary surfactant proteins (SP) are categorized into two groups, namely, hydrophobic surfactant proteins (SP-B and SP-C) and hydrophilic surfactant proteins (SP-A and SP-D). SP-A and SP-D, as innate immune proteins, exhibit antibacterial and antifungal activity [10,11]. The expression of SP genes is regulated by several nuclear proteins, including thyroid transcription factor-1 (TTF-1) and hepatocyte nuclear factor-3β (HNF-3β) [12]. The cellular mechanism, referred to as the heat shock response (HSR), is activated to preserve proteostasis in the presence of factors that lead to proteotoxic stress, such as high temperatures, elevated oxygen levels, heavy metals, and bacterial infections [13,14,15]. Although there is only one ortholog of heat shock factor 1 (HSF1) in invertebrates, such as *Caenorhabditis elegans*, *Drosophila melanogaster*, and yeast, mammals have four distinct HSFs (HSF1–HSF4).

HSF1 is a major regulator of heat shock protein transcription in eukaryotes and has been implicated in several fundamental biological processes, independent of HSR, including metabolism, gametogenesis, development, and aging. It has also been linked to various pathological conditions [14,16,17]. HSF1 is a eukaryotic transcription factor that adjusts the cellular proteostasis system to changing stress loads by inducing the expression of a large set of genes [14,18]. In the presence of stress stimuli, HSF1 binds to its target elements in the genome to induce the transcription of proteases and heat shock proteins (HSPs). In mammals, multiple layers of regulation, cytosolic restriction, and monomerization are inactivated by HSF1, activated by stress with nuclear targeting, and DNA binding [17].

In this study, *A*. *fumigatus* conidia was used to evaluate the cellular immune reaction at the transcription level in A549 human lung epithelial cells. The transcription factors that function during the transcription process were identified. The results indicate that transcription factor HSF1 is an effective therapeutic target for the treatment of a variety of fungal infections.

## 2. Results

### 2.1. Identification of the Conidia Response Region in the SFTPD Promoter

**Figure 1:**
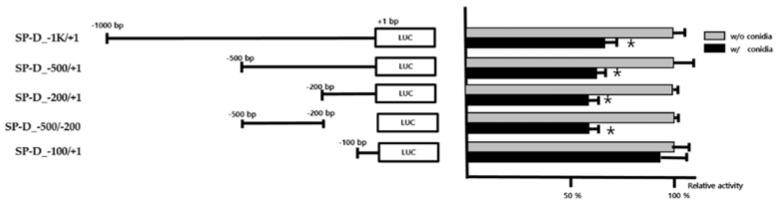
Luciferase activity of the truncated promoter of SFTPD. The schematic illustration on the left side illustrates each truncated promoter construct. The graph on the right shows the luciferase activity of the SFTPD promoter constructs. The promoter activity of each construct is expressed as the percent activity relative to vector-only cells. Histograms represent the mean+/−SD values of three independent transfection experiments with two different plasmid preparations. Significance was determined using Student’s *t*-test. * *p* < 0.05 vs. the group without conidia.

To investigate the mechanisms that regulate SFTPD expression, the 5′-flanking region of SFTPD was dissected into a series of deletion fragments termed SP-D_1k/+1 (−1000 to +1 bp), SP-D_−500/+1 (−500 to +1 bp), SP-D_−200/+1 (−200 to +1 bp), and SP-D_−100/+1 (−100 to +1 bp). A luciferase reporter assay was used to measure the transcriptional activity of the fragments (Figure 1). Comparing the vector-only group with the conidia-treated group, we found that the luciferase activity of SP-D_1k/+1, SP-D_−500/+1, and SP-D_−200/+1 was significantly suppressed in A549 cells exposed to conidia (*p* < 0.05). However, there was no suppression of SP-D_−100/+1 with conidia. Next, we measured the promoter activity devoid of the -200 to −100 bp region (Figure 1). Transcriptional activity of the SP-D_-500/ SP-D_-200 group compared with that of the SP-D_−200/+1 group (*p* < 0.05) was not affected by conidia treatment. This suggests the presence of repressive regulatory elements in the region between positions −200 and −100 bp.

### 2.2. Use of Decoys to Inhibit Conidia-Mediated Suppression of the Promoter Activity

To define a more exact location of transcription factor binding in the promoter region associated with the suppression of SFTPD transcription, we used each decoy oligonucleotide approach to find the specific transcription factor binding site(s) in conidia-treated cells. The decoy oligonucleotides bind to transcription factors and inhibit binding to the corresponding cellular target sequences.

**Figure 2:**
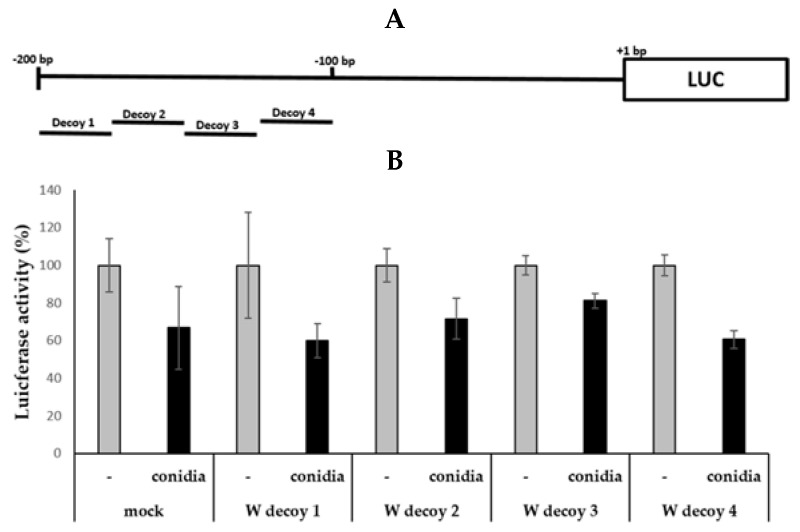
Decoy oligonucleotides corresponding to the conidia response element inhibit the suppression of SFTPD promoter activity. A549 cells were cotransfected with SP-D_−200/+1 constructs in the presence of decoy oligonucleotides using different promoter regions: (**A**) schematic illustration of the location of the decoy oligonucleotide in the promoter region of SFTPD; (**B**) luciferase assay of SFTPD constructs with different decoy oligonucleotides of the SFTPD promoter region. Significance was determined by Student’s *t*-test. * *p* < 0.05 vs. the group without conidia.

We designed the decoy oligonucleotides (25 bp) against four different regions (Table 1). The location of each decoy oligonucleotides is shown in Figure 2A. The luciferase activity levels were significantly increased in the experimental group treated with the Decoy 3 oligonucleotide compared with others groups, and this result suggests that the Decoy 3 oligonucleotide inhibits conidia-induced suppression of SFTPD transcription activity (Figure 2B). Thus, it was shown that one of the transcription factors that can function as a suppressor is expected to bind to a specific region (−150 to −126) in the SFTPD promoter region.

### 2.3. In Silico Analyses

The Decoy 3 oligonucleotide inhibited conidia-induced suppression during SFTPD expression in A549 cells. We identified transcription factor–DNA interaction in this region. The online prediction bioinformatics software, AliBaba2 (http://www.gene-regulation.com/pub/programs/aliba-ba2/index.html. (accessed on 1 October 2019)), was used for in silico analysis. Known transcription factors with binding sites within this sequence were identified by this software.

**Figure 3:**
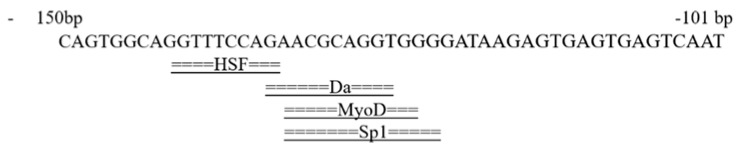
Putative transcription factor binding sites in the promoter region (−150 to −101) of SFTPD according to the AliBaba2 program.

In silico analyses of the SFTPD promoter sequence from −150 to −101 (50 bp) predicted a clustered site containing sites for the transcription factor HSF1 (Figure 3). Other factors binding within this cluster were predicted to be Da, MyoD, and SP-1. These results indicate that HSF1 is a strong candidate for regulating transcription activity in this region from −150 to −126 (25 bp).

### 2.4. Use of a Mutated Decoy Form to Inhibit Conidia-Mediated Suppression of SFTPD Promoter Activity

**Figure 4:**
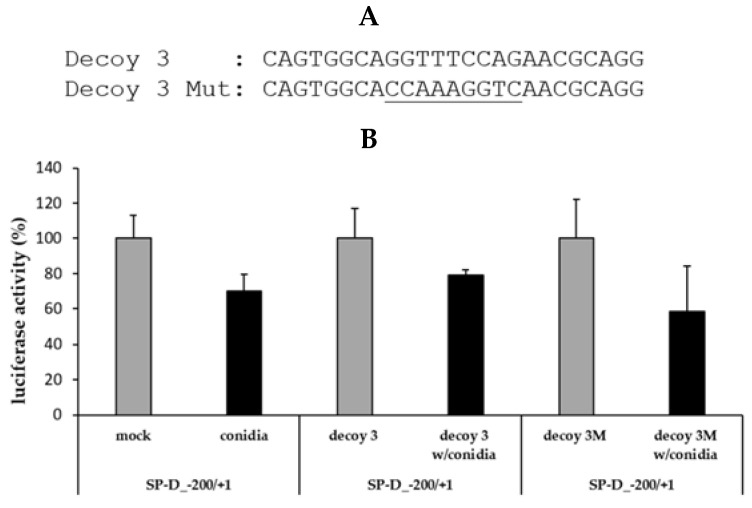
Mutant decoy oligonucleotides corresponding to the HSF1 binding element inhibit the suppression of Decoy 3 oligonucleotide-induced promoter activity: (**A**) sequence of a mutated form of the decoy oligonucleotide of the HSF1 binding site. The mutated sequences in the decoy oligonucleotide are denoted by the underlined letters. (**B**) Luciferase assay with Decoy 3 Mut.

The in silico analysis predicted that HSF1 is a transcriptional repressor located in the −150/−126 region of the SFTPD promoter. To confirm that this region is essential for HSF1-mediated suppression of SFTPD expression, we replaced the nucleotide sequence GGTTTCCAG with CCAAAGGTC in the Decoy 3 oligonucleotide to inhibit HSF1 binding (Figure 4A). We cotransfected this construct with the SFTPD promoter region construct into A549 cells. The SP-D_−200/+1 construct with a Decoy 3 Mut oligonucleotide, which contains an HSF1 mutation binding sequence, exhibited the same promoter activity as the group without a decoy oligonucleotide. Compared with the Decoy 3 oligonucleotide group, the promoter activity with the mutated decoy oligonucleotide was suppressed to a level of 15%, which was identical to the group containing only the construct (Figure 4B). Therefore, the results suggest that HSF1 acts as a suppressor of SFTPD and that the core site required for HSF1-mediated SFTPD promoter suppression resides in the region from −142 to −134.

### 2.5. Conidia Invasion Induces the Binding of HSF1 to SFTPD Promoters

**Figure 5:**
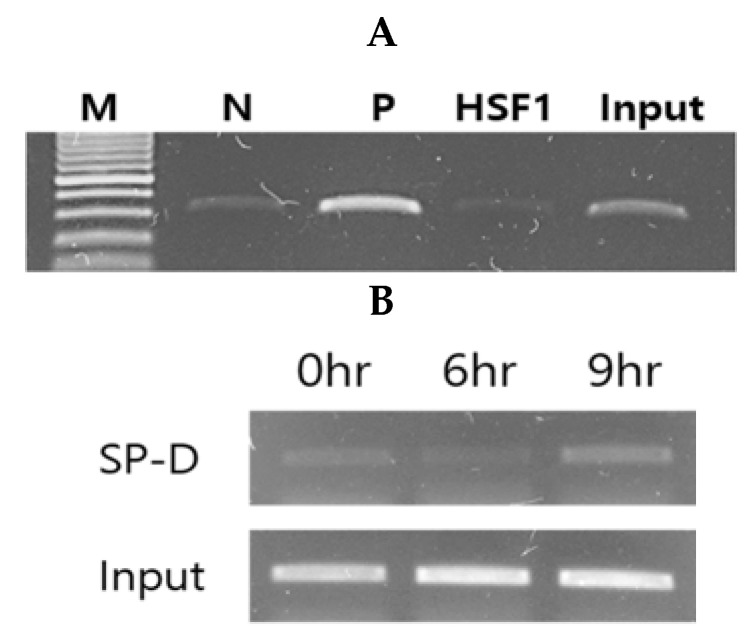
Agarose gel image illustrating the ChIP region of the promoter that binds to SFTPD after immunoprecipitation with specific anti-HSF1 Ab (HSF1 Ab), control IgG, and a positive control in pretreated cells (**A**) and time-dependent response in conidia-treated cells (**B**). The input represents 2% of total cross-linked, reversed chromatin before immunoprecipitation.

ChIP assays were performed to test whether conidia exposure induces binding of HSF1 to the SFTPD promoter region in A549 cells. ChIP lysates were prepared from serum-starved and conidia-treated cells and immunoprecipitated with an anti-HSF1 antibody or a control antibody. As shown in Figure 5A, there was a minimal amount of HSF1 associated with the SFTPD promoters in the serum-starved cells without conidia, whereas conidia treatment (9-h incubation) resulted in the recruitment of HSF1 to the promoters (Figure 5B). These results suggest that HSF1 can bind to the SFTPD promoter in conidia-treated A549 cells in a time-dependent manner.

### 2.6. HSF1 Expression in Conidia-Treated Cells

**Figure 6:**
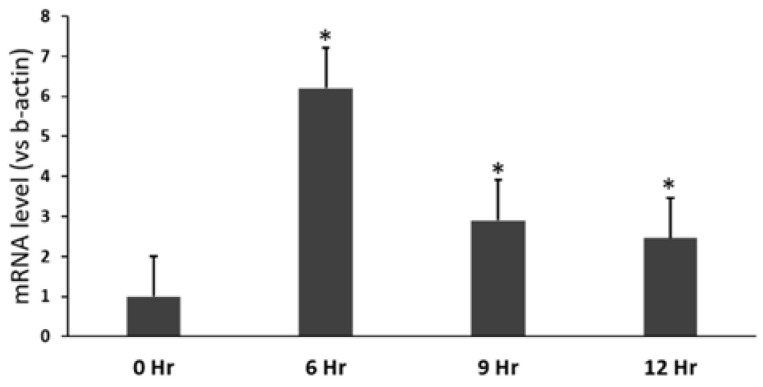
mRNA expression of the HSF1 transcription factor in cells treated with *A*. *fumigatus* conidia. Significance was determined using Student’s *t*-test. * *p* < 0.05 vs. the group without conidia (0 h).

To investigate the change in HSF1 expression during the conidia infection process, we assessed the mRNA levels of HSF1 in *A*. *fumigatus* conidia-infected cells. HSF1 mRNA was highly expressed following a 6-h exposure (Figure 6). This finding indicates that the infection of *A*. *fumigatus* conidia leads to the upregulation of HSF1 in infected cells after a 6-h incubation, and suppresses SFTPD expression.

## 3. Discussion

A previous study found that *A*. *fumigatus* conidia infections caused a decrease in SFTPD expression. Here we found that HSF1 is highly expressed and functions as a negative regulator of the SFTPD expression in *A*. *fumigatus* conidia-infected cells. Aspergillosis represents a significant danger to immunocompromised individuals. When these patients inhale ubiquitously present airborne conidia as *A*. *fumigatus* and *A*. *flavus*, which are the predominant human pathogenic species, fungal invasions can infect the lung [19]. The invasion of *Aspergillus* into the pulmonary arterioles occurs through hematogenic dissemination in approximately one-third of patients with pulmonary aspergillosis. Consequences include thrombosis, hemorrhagic infarctions, and the involvement of distant organs [20,21]. Despite the widespread availability of antifungal drugs, IA mortality is extraordinarily high and ranges from 30% to 90% [6,22,23,24]. Mortality rates up to 40–50% are reported in patients with acute leukemia, and recipients of hematopoietic stem cell transplantation suffer from invasive aspergillosis [25]. In addition to the underlying diseases and immunosuppression issues, the primary determinants of survival include rapid diagnosis and early antifungal treatments [26]. Hence, it is necessary to develop new therapeutics to combat these diseases and improve clinical outcomes for patients.

Serum SFTPD levels in allergic patients with hypersensitive lung disease and in asthmatics are known to increase upon medical diagnosis and decrease following corticosteroid treatment [27]. For patients with idiopathic pulmonary fibrosis, SFTPD levels in the blood increase, and elevated SFTPD levels are detected in patients with interstitial pneumonia during collagen vascular disease and pulmonary alveolar proteinosis [28]. SFTPD exerts several effects on the regulation of the innate immune response in the lung [9]. For example, SFTPD inhibits the dissemination of infectious microbes by inhibiting their behavior, for example, through agglutination and growth suppression [29]. Moreover, SFTPD enhances the elimination of microbes by assisting with phagocytosis by macrophages. It also binds to pattern recognition receptors, such as toll-like receptors CD14 and MD-2, to regulate the inflammatory responses [30]. Thus, SFTPD is relevant to the host defense against *A. fumigatus* [31].

Fungal adhesion of the epithelium was shown to decrease, and fungal clearance by neutrophils was shown to increase in the presence of SFTPD. SFTPD binds to and agglutinates *A. fumigatus* conidia in a calcium-dependent manner. SFTPD-opsonized, dormant conidia are phagocytosed efficiently by human monocyte-derived macrophages, which promote the secretion of pro-inflammatory cytokines [32,33]. Additionally, SFTPD induces the production of cytokines and chemokines to increase clearance of bacterial, viral, and fungal pathogens. The protective roles of SFTPD during bacterial and viral infection have been thoroughly investigated [34]. Inflammation induced by *A*. *fumigatus* is related to increased expression levels of the SFTPD protein [35]; however, its expression during fungi infections has not been well characterized even though many fungal disease studies have been conducted with *A*. *fumigatus*. There is a direct interaction between NFATc3 and TTF-1 to activate SFTPD expression cooperatively during transcription [36]. C/EBP elements can also interact with the promoter region to regulate SFTPD expression [37]. During transcription, SFTPD expression levels are regulated by AP-1 proteins in pulmonary epithelial cells [38]. Stress is sometimes good for the body’s self-protection, although stress occasionally renders the body susceptible to disease [39].

Heat shock factor 1 (HSF1) forms oligomers in the nucleus and increases affinity for binding to what are known as heat shock elements under stress conditions [40]. HSF1 is an elementary factor of the cellular stress response, protein translation, and cellular metabolism [41], which maintains proteostasis by regulating heat shock proteins such as HSP90 [42]. HSF1 also controls heat shock, or proteotoxic stress, transcriptionally, thereby safeguarding the proteome [17].

Our results indicated that HSF1 is also increased in *A*. *fumigatus* conidia-infected cells. More importantly, HSF1 as a transcription factor serves as a negative regulator of SFTPD expression, and an increased SFTPD level helps cells protect from an infectious conidia invasion by *A*. *fumigatus*. These findings clearly indicate that the HSF1 transcription factor is a good indicator of *A. fumigatus* conidia infection via SFTPD and that it represents a candidate for use in aspergillosis treatment.

## 4. Materials and Methods

### 4.1. Chemicals and Cell Culture

All chemicals were obtained from Sigma-Aldrich (St. Louis, MO, USA). All cells were incubated in RPMI 1640 medium supplemented with 10% FBS and antibiotics and maintained in a humidified incubator at 37 °C and 5% CO_2_. The medium was changed every 3 days. A549 cells were grown in six-well tissue culture plates for 18 h, and the cells were incubated with conidia (5 × 10^7^ spores) for the experiments.

### 4.2. Vector Construction and Transfection

The PCR reactions consisted of an initial denaturation at 94 °C for 5 min and 35 cycles at 94 °C for 1 min, 55 °C for 1 min, 72 °C for 1 min, and 72 °C for 10 min. The sequence-confirmed PCR products were subsequently cloned into pGL4.14-lac2 (Promega, Madison, WI, USA) at the KpnI and XhoI sites for the luciferase assays. A549 cells were transfected with pGL4.14-lac2 containing various SFTPD promoter regions using Lipofectamine 2000 reagent (Invitrogen, Carlsbad, CA, USA) according to manufacturer’s instruction. After 24 h of transfection, we analyzed the promoter activity with a luciferase assay.

### 4.3. Decoy Assay

Decoy oligonucleotides corresponding to the conidia response element inhibited the suppression of SFTPD promoter activity. A549 cells were cotransfected with SFTPD constructs in the presence of wild-type or mutant decoy oligonucleotides. The wild-type decoy oligonucleotide attenuated the conidia-induced suppression (reported as %) of the reporter gene construct containing a conidia response site in A549 cells. These results were from at least two independent transfection experiments performed in triplicate.

### 4.4. Luciferase Assay

Firefly and Renilla luciferase activities were measured by dual luciferase assays (Promega) and then normalized to Renilla luciferase activity. The luciferase activity of the SFTPD promoter constructs in A549 cells was measured. The promoter activity of each construct was expressed as the percent activity of vector-only treated cells. Histograms represent the mean values of three independent transfection experiments with two different plasmid preparations.

### 4.5. Prediction of the Transcription Factor

A portion of the 50-bp SFTPD promoter region was subjected to computer-aided analyses using the Alibaba2.1 net-based transcription factor binding site (TFBS) search tool (http://www.gene-regulation.com/pub/databases.html (accessed on 1 October 2019) and the Transfac 7.0 database. Binding sites identified using TRANSFAC are used for this purpose, whereas AliBaba2 is currently the most specific tool for predicting such sites.

### 4.6. Chromatin Immunoprecipitation Assays

Chromatin immunoprecipitation assays (ChIP) assays were performed with the ChIP-IT enzymatic kit (Cell Signaling, Danvers, MA, USA). Briefly, cells were cross-linked with 1% formaldehyde, and the chromatin was sheared with an enzymatic shearing cocktail. Chromatin-containing DNA fragments with an average size of 500 bp were immunoprecipitated with an anti-HSF1 monoclonal antibody (Santa Cruz Biotechnology, Dallas, TX, USA) with rabbit IgG as a negative and histone H3 rabbit mAb as a positive control. DNA fragments were amplified using primers designed around the HSF1 binding sites in the SFTPD promoter region.

The protein–DNA complexes were incubated with protein A beads, and cross-links were reversed by incubating at 65 °C. DNA was recovered by phenol-chloroform extraction, precipitated with ethanol, and then subjected to semiquantitative PCR analysis. The primer sets were as follows: 5′-CTGGACTCCTGATTTCTAGAC-3′ and 5′-AGCCTTTGCTTGGCAGGCA-3′ (−300 to +1, PCR product 300 bp). PCR products were resolved on a 2% agarose gel and visualized by ethidium bromide staining.

### 4.7. Nucleic Acid Manipulation and Quantitative RT-PCR

Total RNA (2 µg in a total volume of 20 µL) was used for reverse transcription. Quantitative RT-PCR was performed according to the manufacturer’s instructions (Qiagen, Hilden, Germany) using a Rotor-Gene Q device (Qiagen). Each run was assayed three times with 2× qPCR SYBR Green Mix (Doctor Protein, Seoul, Korea) and 100 nM of each primer. Table 2 lists the primer sets that were used to detect HSF1 and *β*-actin [43]. The one-step qRT-PCR conditions were as follows: 42 °C (40 min) for reverse transcription and 95 °C (15 min) for one cycle, followed by 95 °C (30 s) and 55 °C (30 s) for 40 cycles. The 2^−ΔΔ*C*t^ method was used to calculate the Ct value for each gene, and target gene expression levels were normalized to that of the β-actin housekeeping gene [44].

## Figures and Tables

**Table 1:** List of decoy oligonucleotide sequences.

Decoy	Sequence (5′–3′)
1	GATAGTGTTCTGCAAACTATAGTGA
2	TGGATGTGCATAGTATTGTGAATAT
3	CAGTGGCAGGTTTCCAGAACGCAGG
4	TGGGGATAAGAGTGAGTGAGTCAAT

**Table 2:** List of the sequences of primers for RT-PCR.

Genes		Sequence (5′–3′)
HSF1	Sense Antisense	GAACAGCTTCCACGTGTTGC TCGATGTGGACCACTTTCCG
β-actin	Sense Antisense	GCCAACACAGTGCTGCTCGG TACTCCTGCTTGCTGATCCA
